# Review paper on WPW and athletes: Let sleeping dogs lie?

**DOI:** 10.1002/clc.23399

**Published:** 2020-06-27

**Authors:** Lisa W.M. Leung, Mark M. Gallagher

**Affiliations:** ^1^ Cardiology Clinical Academic Group St. George's University Hospitals NHS Foundation Trust, St. George's University of London London UK

**Keywords:** athletes, cardiac screening, catheter ablation of accessory pathways, pre‐excitation, sudden cardiac death, Wolff‐Parkinson‐White syndrome, WPW

## Abstract

Accessory pathways are present in 1 in 300 young individuals. They are often asymptomatic and potentially lethal arrhythmias may be the first presentation. During long‐term follow‐up, up to 20% of asymptomatic individuals with pre‐excitation go on to develop an arrhythmia and the absence of traditional clinical and electrophysiological high‐risk markers does not guarantee the “safe” nature of an accessory pathway. The widespread availability of permanent cure for the condition at low risk by catheter ablation, creates an incentive to screen for accessory pathways with a 12‐lead ECG, particularly in individuals who are perceived to be at increased risk, such as athletes and high‐risk professions. We review the existing literature on the assessment and management of accessory pathways (Wolff‐Parkinson‐White [WPW] syndrome) and discuss its implications for the young athletic population.

## 1 INTRODUCTION

Accessory pathways (Wolff‐Parkinson‐White Syndrome [WPW]) are often asymptomatic and potentially lethal arrhythmias may be the first presentation. Due to the requirement for cardiac screening with a 12‐lead ECG in most athletic disciplines and associated sporting organizations, a pre‐excitation pattern may be identified in otherwise healthy individuals. This review aims to address in a systematic way the assessment and management of WPW, with particular emphasis on the athletic population.

## 2 METHODOLOGY

For the purpose of this review, a search for available literature between 1930 and 2020 was conducted, using PUBMED, using the key words: Wolff‐Parkinson‐White, pre‐excitation, athletes, accessory pathway, sudden cardiac death, life‐threatening events, screening, and ablation. A summary of the key studies included in the review is outlined in Table 1.

**TABLE 1:** Key studies reviewed in this article

Key studies reviewed	Year of publication and type of study	Number and type of patients studied	Summary of findings
Garg et al.^42^	2017. Retrospective study of outcomes after WPW catheter ablation in the United States over a period of 14 years from 1998 to 2011.	2329 patients had WPW catheter ablation in this time period according to ICD coding.	Catheter ablation is a safe procedure with low morbidity and mortality risk. Cardiac tamponade occurred in 0.9% of cases; complete heart block occurred in 1% of cases.
Liberman et al.^45^	2014. Retrospective single‐centre study of all anteroseptal AP cases who underwent cryoablation during the period of 2005‐2012.	70 patients <21 years of age had anteroseptal AP and received cryotherapy ablation treatment.	Septal pathway ablation in young patients using cryotherapy had no cases of complete AV block in this study.
Etheridge et al.^51^	2018. Retrospective paediatric multi‐centre study.	912 subjects <21 years old from general population, diagnosed with WPW from EP study.	Life‐threatening events may occur despite the absence of high risk features from EP study or prior symptoms. 10% of these events occurred during exercise.
Pappone et al.^49^	2003. Prospective randomised trial of asymptomatic WPW patients.	Asymptomatic WPW patients. 224 were eligible for the study with final study participants of 37 randomised to ablation group and 35 randomised to control group.	1 control group patient had an aborted VF cardiac arrest. The 5 year Kaplan‐Meier survival estimates of incidence of arrhythmic events were 7% in ablation group versus 77% in controls (*P* < .001).
Pappone et al.^48^	2012. Prospective long‐term follow‐up study of those who had EP study for WPW.	8575 patients with symptomatic WPW from AVRT had EP study. 369 patients declined ablation treatment after EP study and had long‐term follow up.	No cases of mortality were recorded in those who refused ablation in this study. 1.1% of the non‐ablation cohort had a life‐threatening event during the 5 year follow‐up period. Short pathway anterograde ERP is a robust marker of risk.
Malhotra et al.^29^	2018. Retrospective study of a nationwide structured cardiac screening program of elite footballers.	11 168 elite level football players; 95% male.	Based on this study, the incidence of SCD was at 6.8 per 100 000 athletes. 26 cases of pre‐excitation pattern were found; 24 were ablated. Two followed up clinically and no cases of WPW related death or aborted SCD were confirmed. All 26 resumed their normal competitive athletic career.
Finocchiaro et al.^6^	2017. Retrospective review of cardiac pathology database of all SCD cases received from 1994‐2014.	3684 cases of SCDs found 19 cases with a prior clinical diagnosis of WPW before death.	Key findings of SCD deaths with prior WPW ECG patterns include; deaths may occur at rest and without prior symptoms. Deaths may occur after the 4th decade. Co‐morbidities existed which increased risk of AF.
Borregaard et al.^52^	2015. Retrospective cohort single‐centre study of WPW patients who had RF ablation.	362 WPW patients who had catheter ablation for WPW.	Post ablation follow up reveals that mortality risk is similar to background population and confirms that there are no long‐term adverse effects of ablation treatment in this cohort. Interestingly, the incidence of AF later on in life was higher in the post‐ablation WPW group.

## 3 HISTORY AND TERMINOLOGY

Drs Louis Wolff, John Parkinson, and Paul Dudley White described what they believed was an association between left bundle branch block and supraventricular tachycardia.^1^, ^2^ It was not a blocked bundle branch but broadening of the QRS due to excitation reaching the ventricle before conduction could reach it through the His‐Purkinje system. This pre‐normal excitation, or pre‐excitation of the ventricular myocardium was via an accessory pathway, an abnormal electrophysiological connection between the atria and the ventricles.

An accessory pathway provides an additional route for conduction from atrium to ventricle and vice versa, bypassing the His‐Purkinje system and AV node; it is therefore often also called a “bypass tract.” The presence of an accessory pathway leaves the patient vulnerable to two forms of arrhythmia: re‐entrant arrhythmias due to the second route of conduction and accelerated conduction of atrial arrhythmias because the pathway bypasses the protection of the AV node. Pre‐excited atrial arrhythmias, particularly atrial fibrillation (AF) can be lethal. The atrial fibrillation cycle length (CL) is variable and short, typically between 150 and 250 ms. But the atrial rate of up to 300 beats per minute can, in some cases, be transmitted directly to the ventricles producing ventricular fibrillation and sudden cardiac death (SCD).^3^, ^4^, ^5^, ^6^

The prevalence of WPW in the general population is estimated at 0.1 to 4.5 per 1000 individuals.^7^, ^8^, ^9^ It is usually an isolated abnormality in an otherwise normal heart in a healthy person. A small minority occur as part of structural congenital or genetic cardiac conditions, notably Ebstein's anomaly or glycogen storage disorders.^10^, ^11^, ^12^, ^13^ The management discussed in this article may not necessarily apply to those with these underlying conditions. Patients with these underlying conditions may present in various ways including, familial cascade screening, cardiac imaging to investigate symptoms or incidentally in cases where investigations were requested for other clinical reasons.

Most individuals with pre‐excitation never experience an arrhythmia.^14^ Accessory pathways often present in the teenage years or early adult life. They rarely produce arrhythmias in‐utero and only occasionally in infancy or early childhood. Because of the potential for lethal arrhythmias and the possibility to be corrected safely and permanently, pre‐excitation has an importance disproportionate to its relative rarity. The great majority of patients do not require long‐term follow after treatment by ablation, and can resume normal activity including intense exercise. It is therefore important that the physician recognize the condition and refer individuals for appropriate evaluation and treatment with an appropriate level of urgency.

## 4 ANATOMICAL AND ELECTROPHYSIOLOGICAL FEATURES

Accessory pathways can be found at any point around the AV ring: left lateral locations are most common, septal and right sided ones slightly less so; they have even been described in the aorto‐mitral continuity. A small proportion run from the compact AV node to the ventricle via the fascicle or directly (called nodofascicular and nodoventricular pathways, respectively) and a few have decremental conduction properties like the tissue of the AV node (Mahaim fibres), but most are just strands of myocardium that traverse the AV groove and have conduction properties similar to atrial myocardium.^15^, ^16^, ^17^, ^18^ This structure can exhibit varied electrophysiological properties, probably in part due to variable branching and arboreous features.

The most important electrophysiological properties of a pathway are its anterograde effective refractory period (RP) and its directionality.^19^, ^20^ Approximately half of accessory pathways conduct in one direction only, most commonly from ventricles to atria (retrogradely). A pathway that conducts only anterogradely can produce pre‐excitation in AF, but seldom supports a regular re‐entrant arrhythmia; a pathway that conducts only retrogradely cannot accelerate the conduction of an atrial arrhythmia, so presents little danger apart from symptomatic atrio‐ventricular reciprocating tachycardia (AVRT). Danger is a product of the anterograde RP of a pathway: A short RP means that the pathway can conduct successive depolarizations at short intervals, therefore risking ventricular activation during AF at a rate fast enough to trigger ventricular fibrillation.

As the electrophysiological property and behavior of an AP may vary, the terms manifest, latent and concealed have been used to describe and clarify this. A *manifest* accessory pathway is one that conducts anterogradely and often can conduct bi‐directionally (both anterograde and retrograde). A *concealed* AP cannot conduct anterogradely and so there is no evidence of pre‐excitation on the ECG. The absence of anterograde conduction makes such a pathway incapable of causing sudden death by pre‐excited AF. *Latent* pre‐excitation refers to anterograde pathway conduction unmasked by certain conditions of the atria and/or the AV node but not seen on the ECG at other times because normal conduction reaches the ventricles before conduction via the pathway. Delaying conduction via the AV node, for example by administering adenosine, can reveal the pathway; pre‐excitation can also be unmasked by premature atrial complexes or by the occurrence of AF.

## 5 WPW AND THE ATHLETE: WHAT'S DIFFERENT?

In classical times, Pheidippides' death after the battle of Marathon was not questioned;^21^ having apparently run 84 km in a day, his subsequent death seemed unremarkable. Now, sport has become more varied, extreme and often over a prolonged duration of time in a person's lifetime, with increasing numbers of people who identify themselves as veteran athletes emerging. The effect of decades of sporting participation on a person's physiology and development of pathophysiological conditions is incompletely understood.^22^

Wolff‐Parkinson‐White accounts for 1% of SCD in the athletic population based on a long‐term registry but this is likely to be an underestimate due to lack of ECG screening.^23^ Diagnosing WPW as a cause of sudden death is difficult; autopsy examination of the heart cannot reliably identify the existence of accessory pathways as this would require extensive dissection of the atrioventricular groove, a time‐consuming process that is not included in a standard autopsy. As such it is possible that a proportion of SCDs secondary to WPW related arrhythmias may be attributed to a structural normal heart also referred as Sudden Arrhythmic Death Syndrome (SADS), which is recognized as a common cause of SCD in contemporary studies in young and athletic individuals.^24^, ^25^, ^26^

Athletic activity accentuates the risk of pre‐excitation related SCD; in one survey, two thirds of the study population suffered from an aborted VF cardiac arrest during exercise or under emotional stress^27^; in the more recent Italian registry, exercise related events remained high at 36%.^20^ Even in sedentary individuals with pre‐excitation, exertions of daily life may precipitate an arrhythmia. Exercise‐related adrenergic activation accelerates AV nodal conduction in both directions, increasing vulnerability to re‐entrant tachycardias and the rate of the tachycardia induced. It may therefore increase the risk of a regular tachycardia disorganizing to AF and precipitating ventricular fibrillation.

Participation in organized athletics presents an opportunity, as screening is used to exclude a variety of conditions, and pre‐excitation can be detected and treated as a result. Because athletic training and competitions are organized by schools, societies and companies, a duty of care is created that involves organizations with the logistical capacity to deliver screening and alongside this be sensitive to the associated ethical and legal considerations.^28^

## 6 SCREENING FOR ATHLETES

A study by Malhotra et al.^29^ looked at the outcomes of cardiac screening in elite football players and found that pre‐excitation was picked up in 26 cases out of a total of 11 168 who underwent screening at the age of 16.4 ± 1.2 years. None of these suffered from a life‐threatening event: 24 cases were ablated and resumed playing and the other two had low risk features and continued to compete with no adverse events recorded.

Screening for the conditions that predispose to SCD in athletes often includes a resting 12‐lead ECG alongside a focused history and a clinical examination. However, the screening protocol can vary depending on the type of organization providing it. In the case of manifest pre‐excitation, the diagnosis derives from the ECG. Good history‐taking and clinical judgement remains important not only for risk stratification purposes, but also because symptoms may be the only evidence of latent or concealed pre‐excitation. The symptoms that are most strongly suggestive of arrhythmias are recurrent syncope, pre‐syncope and sustained palpitation, particularly when associated with exercise. To make the screening process more challenging, athletes may not report or under report symptoms for fear of interruption to their training, funding or other impact to their career.

**FIGURE 1:**
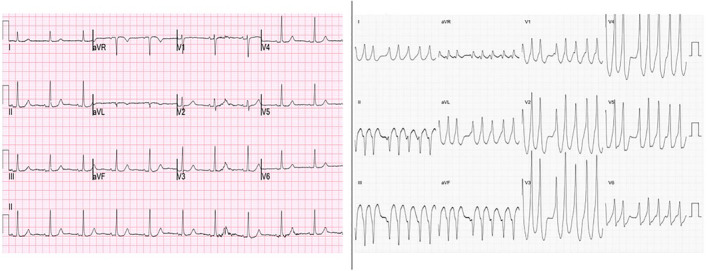
An example of latent pre‐excitation: a 12 lead ECG in sinus rhythm and another documenting preexcited AF in the same patient

The electrocardiographic hallmarks of pre‐excitation are a PR interval shorter than 120 ms and a slurred upstroke to the QRS complex known as a delta wave for its shape. The degree to which pre‐excitation is evident depends on the relative time taken for a sinus beat to conduct to the ventricle via the accessory pathway and via the conduction system. Activation takes so long to reach a pathway in a left lateral location that AV nodal conduction will often beat it, masking its presence. A patient with WPW may therefore have a perfectly normal ECG even with a rapidly conducting pathway with a short anterograde RP (Figure 1).

The pattern of the pre‐excitation on the ECG can be dynamic.^30^ The existence of pre‐excitation on an ECG may be subtle and easily missed. The ECG must be analyzed systematically: the PR interval measured precisely, the onset of the QRS complex inspected for slurring, the QRS axis and transition defined. Automated analysis provides a reasonably reliable way of supporting this process, but confirmation by an expert cardiologist is still required. Any patient showing abnormality of any of these indices must be considered for possible pre‐excitation. However, a short PR interval, in isolation, is known to be a physiological finding in a young person or athlete, representing rapid AV nodal conduction. In electrophysiological study, there is a reference range for normal AH intervals (50‐120 ms)^31^ in healthy subjects and this is dependent on autonomic efferent output.

## 7 POST‐SCREENING INVESTIGATION

### 7.1 Non‐invasive tests

When the surface ECG is inconclusive, an ambulatory ECG may help to clarify whether pre‐excitation is present. Over the course of any 24‐h period, conduction via the AV node varies, and periods of slower conduction will make pre‐excitation more evident. Atrial premature beats may provide even greater accentuation of pre‐excitation, partly by their prematurity which elicits slower conduction via the AV node due to its decremental conduction properties, partly due to the site of origin: Ectopics arising in the left atrium reach the atrial insertion of a left sided pathway before reaching the AV node, making the pathway conduction more evident.

The ambulatory ECG may also help to clarify the risk associated with an accessory pathway by revealing arrhythmias mediated by it and by providing a measure of the AERP. Most re‐entrant arrhythmias produce symptoms, but in a few cases asymptomatic arrhythmias are revealed. Information about the anterograde RP is difficult to extract because the temporal resolution of Holter systems is less than that of 12‐lead ECG systems and the quality is often inferior. In some cases, particularly when atrial premature beats are present and represent a spread of coupling intervals, useful information may be derived.

The abrupt and complete loss of pre‐excitation during an exercise treadmill test provides a good measure of the anterograde RP of a pathway and therefore of the risk it produces.^32^, ^33^, ^34^ In the real world, the phenomenon can only be seen in a minority of pediatric and adult cases and that there is a wide discrepancy in observer interpretation. The difficulty arises from the normal tendency for conduction via the AV node to accelerate as the sinus rate increases with exercise. This produces a progressive encroachment of the sharp part of the QRS upstroke produced by the His‐Purkinje system on the delta wave produced by the AP, masking the existence of pre‐excitation.

**FIGURE 2:**
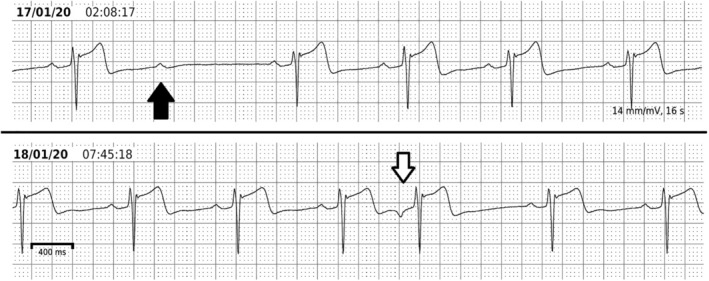
Ambulatory ECG findings that rule out pre‐excitation. This athlete was investigated because of a relatively short PR interval (115 ms) with non‐specific broadening of the QRS complex on a routine 12‐lead ECG. A 5‐day, single derivation recording was performed. This shows lengthening of the PR interval to 200 ms during sleep without alteration of the QRS complex. There are also instances of second degree AV block during sleep (black arrow) and atrial premature beats which are conducted at a longer PR interval without alteration in the QRS complex. All of these features indicate that either the patient has no pathway capable of anterograde conduction or that the AERP of the pathway is longer than the sinus cycle present at the time of the second degree AV block, which was over 1 s

In some cases, the surface ECG is suggestive of pre‐excitation but not diagnostic. If the ambulatory ECG does not clarify this issue (Figure 2), injection of adenosine can usually do so. A sufficient bolus of adenosine injected into a large vein will cause transient block of AV nodal conduction; if a pathway with anterograde conduction is present, it will be unmasked by this.^35^ If one or more P waves are blocked from AV conduction, then it is safe to conclude that no clinically dangerous accessory pathway is present.

Intermittent pre‐excitation is not considered to be a robust sign of a low risk pathway although there is some evidence that intermittent pathway activity is associated with a long anterograde effective refractory period.^36^ Recent studies and the 2019 ESC guidelines support the fact that intermittent pre‐excitation is a weak and flawed sign of low risk: >20% of these cases go on to have EPS confirmation of AP ERP <250 ms.^37^, ^38^

### 7.2 Invasive tests

The diagnostic electrophysiological study (EPS) provides a robust measure of the anterograde effective refractory period of any pathway and is the best verified method of risk stratification.^9^, ^20^, ^39^A refractory period of <250 ms is a reproducible indicator of a high‐risk pathway. In the electrophysiology lab, AF can easily be induced and the shortest R‐R interval in AF can be measured; the occurrence of intervals <250 ms is also another high‐risk feature. The more definitive risk stratification of invasive EPS must be weighed against the minor risks involved, such as 1% chance of hematoma at the access site which is rarely more than a transient nuisance. It is also worth noting that in practice, the diagnostic EPS is almost always used as a prelude to curative catheter ablation rather than as a stand‐alone procedure.

## 8 TREATMENT

### 8.1 Medical therapy

Offering blanket suppression of arrhythmic potential by long‐term medication is inappropriate due to the very low rate of clinical success, the long‐term risk of adverse effects including lethal arrhythmias provoked by prolongation of the QT interval or slowing of conduction, and interference with the athlete achieving his/her athletic potential. In general, regular anti‐arrhythmic medication should only be considered in symptomatic patients as short‐term instruments of symptom control if ablation is not immediately available,^40^ or to those who refuse ablation. Sometimes, there may be specific reasons for declining ablation including the pathway being close to the conduction system resulting in a high risk of complete AV block.

### 8.2 Ablation

**FIGURE 3:**
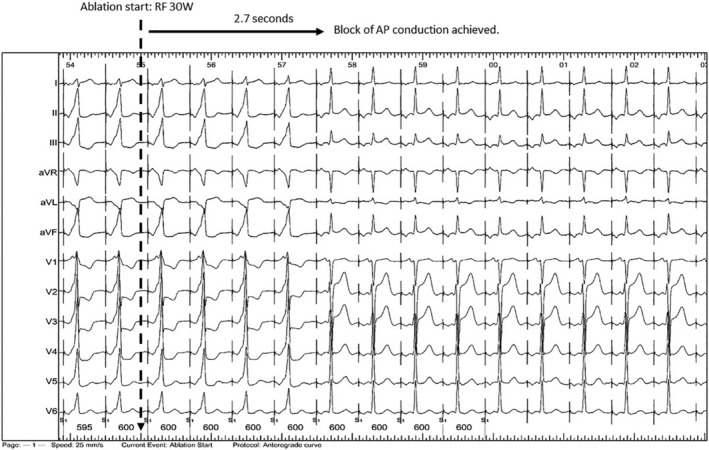
Treatment of WPW. At the left of the figure, the patient has obvious pre‐excitation. This had been present from birth and had caused a near‐arrest due to pre‐excited atrial fibrillation. Radiofrequency energy begins at the time indicated, and <3 s later the pathway becomes blocked. The patient now has a normal heart, and it will remain so for life

The cure rate of accessory pathways by ablation now approaches 100%, with more than 90% of patients obtaining permanent cure on a first attempt (Figure 3).^36^, ^38^ Ablation is typically performed under local anesthesia for adult patients, often under general anesthesia for children. Venous vascular access and cannulation is almost always via the femoral vein(s) and may be guided by ultrasound.^41^ The procedure involves a baseline EPS to determine the electrophysiological properties of the AP and define its location. This provides the basis for a risk‐benefit calculation to decide whether to proceed to ablation, and which method to use: Radiofrequency (RF) is usually chosen for its rapidity and reliability, but cryotherapy may be chosen. Successful ablation is achieved within seconds of the start of RF delivery and is indicated by disappearance of pathway conduction. It is usual to observe for a waiting period of 20 to 30 min and to perform adenosine testing.

The safety profile of catheter ablation treatment has been documented by Garg et al.^42^ They reviewed WPW catheter ablation procedures performed in the United States over a period of 14 years and found that among 11 601 ablations performed, there was no case of in‐hospital mortality associated with the procedure. Cardiac tamponade occurred in 0.9% of cases. Complete heart block was seen in 1% of this series of ablations performed in the period of 1998 to 2011 but is only relevant to a small and well‐defined minority of pathways located close to the conduction system.

Septal pathways accounted for approximately 25% of a series of 1969 cases undergoing EPS.^43^ Septal pathways may be so close to the conduction system that there is a risk of causing complete AV nodal block during ablation. In children, this risk has been quoted at 10% for mid‐septal pathways using RF energy but more recent literature in the same population reports a risk of <1% using cryotherapy.^44^, ^45^, ^46^

Compared to RF, cryotherapy has the advantage of partial reversibility: Tissue that is cooled ceases to conduct long before it sustains permanent injury. For this reason, cryotherapy is often chosen when the pathway lies within 5 mm of the His bundle or the compact AV node.^47^ Cryotherapy also has the advantage of being painless, whereas RF can be moderately painful in some locations. Ablation technology has advanced greatly since the period covered by the Garg series, but no substantial series is available to determine the effect on safety.

An EPS study with a view to ablation is recommended in all symptomatic individuals.^38^ A detailed long‐term follow up study of 369 WPW patients who presented with symptoms but declined catheter ablation, was conducted by Pappone et al.^48^ There were no deaths, but four patients (1.1%) suffered from hemodynamic collapse with pre‐excited AF or VF and a further 25 (6.8%) had pre‐syncope or syncope. The study demonstrated that almost 2% of symptomatic patients suffer life‐threatening arrhythmias for each year that ablation is delayed, confirming the routine use of ablation as first line therapy.

When pre‐excitation is identified in a young and *asymptomatic* athletic individual, the question asked is whether there is a case for completely non‐invasive management. Risk assessment of the AP must take place before the athlete can be deemed “cleared” in preparticipation. An EPS ± ablation is considered first line investigation in the majority of cases and a referral to cardiac electrophysiology is therefore recommended in all, including asymptomatic individuals.

A small randomized controlled trial on asymptomatic pre‐excitation found that the group who received ablation had fewer arrhythmic events compared to the control group (7% vs 77%; *P* < .001) over a period of 5 years.^49^ One patient in the control group suffered from an aborted VF cardiac arrest. A more recent analysis from the same group demonstrated that the prognosis of the Wolff‐Parkinson‐White syndrome depends on intrinsic electrophysiological properties of AP rather than symptoms, further supporting the need for an EPS study for risk stratification of these individuals with ablation performed during the same procedure in many to improve prognosis.^20^

A recent systematic review of the management of asymptomatic children with pre‐excitation found that management for this age group follows similar principles to those in adults. Raposo et al.^50^ found that the cut off age of 8 years or greater was adhered to as recommended under 2012 pediatric guidance. Identification of the shortest pre‐excited R‐R interval (SPERRI) <250 ms was still the best predictor for risk stratification. In pediatric cases, the adjunctive use of isoproterenol to enhance the sensitivity of risk stratification was common as the procedures are generally performed under general anesthesia. The review confirmed that in asymptomatic children, prophylactic ablation is offered if there is a finding of a short anterograde RP or short RR intervals in AF. The presence of multiple pathways was also a factor taken into account.

Etheridge et al. reported on 96 children with WPW who had a life‐threatening event.^51^ The majority did not have prior symptoms. 10% of events occurred whilst participating in sporting activities. They highlight the limitations of all modes of risk stratification: 25% of their series of patients who had suffered a life‐threatening event had neither an inducible tachycardia nor any other definable risk factor, possibly due to use of anesthesia during EPS or due to a failure to define the anterograde RP of the pathway because of atrial refractoriness. The authors concluded that for both young athletic individuals and children with an accessory pathway, life‐threatening events may occur without any prior symptoms, and without traditional markers of high risk on EPS and exercise restriction alone does not keep athletes or children safe. They recommend the adoption of a low threshold for ablation in children with pre‐excitation.

### 8.3 What the 2019 guidelines add

**FIGURE 4:**
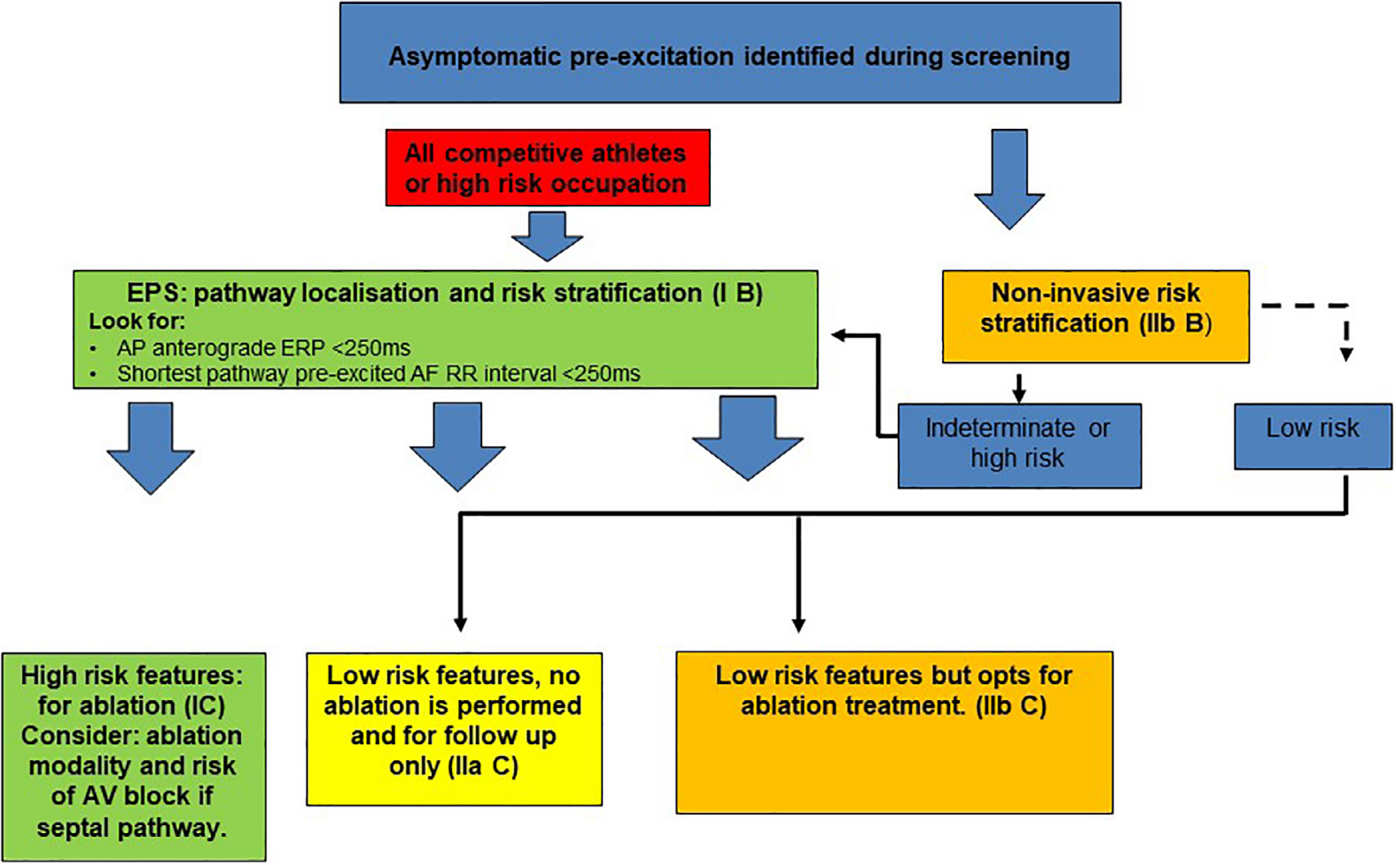
Risk stratification for asymptomatic pre‐excitation. Redrawn and modified from fig. 22, section 11, 2019 ESC Guidelines (Reference 38)

The 2019 ESC guidelines for the management of patients with supraventricular tachycardia provide a good summary of the acute and long‐term management of WPW.^38^ On asymptomatic pre‐excitation, it confirms that formal follow up is required as 20% of these patients will go on to develop symptoms. Importantly, the guidelines establish EPS as the first‐line tool for risk stratification in all individuals with pre‐excitation, irrespective of symptoms, class I recommendation for competitive athletes or high‐risk occupations and IIa recommendation for all others. With data from a Danish registry,^52^ it is also able to clarify that right antero‐septal APs may be linked to future development of AF and heart failure and that mortality risk associated with this AP location was higher even in the older age group of >65. This association had not previously been recognized. A summary of risk stratification for asymptomatic pre‐excitation, adapted from the 2019 Guidelines, is outlined in Figure 4.

## 9 CONCLUSIONS

Cardiac screening in the young and athletic cohort may uncover pre‐excitation. Evidence of an accessory pathway is significant even if the individual is asymptomatic at the time. The condition is potentially lethal if unrecognized but is amenable to a safe and permanent cure once diagnosed.

## Supporting information


**Figure S1**

**Figure S2**
