# Supplementary figures and images for: Review paper on WPW and athletes: Let sleeping dogs lie?
Source: Clin Cardiol. 2020 Jun 27;43(8):897–905. doi: 10.1002/clc.23399 (PMC7403723; doi:10.1002/clc.23399)

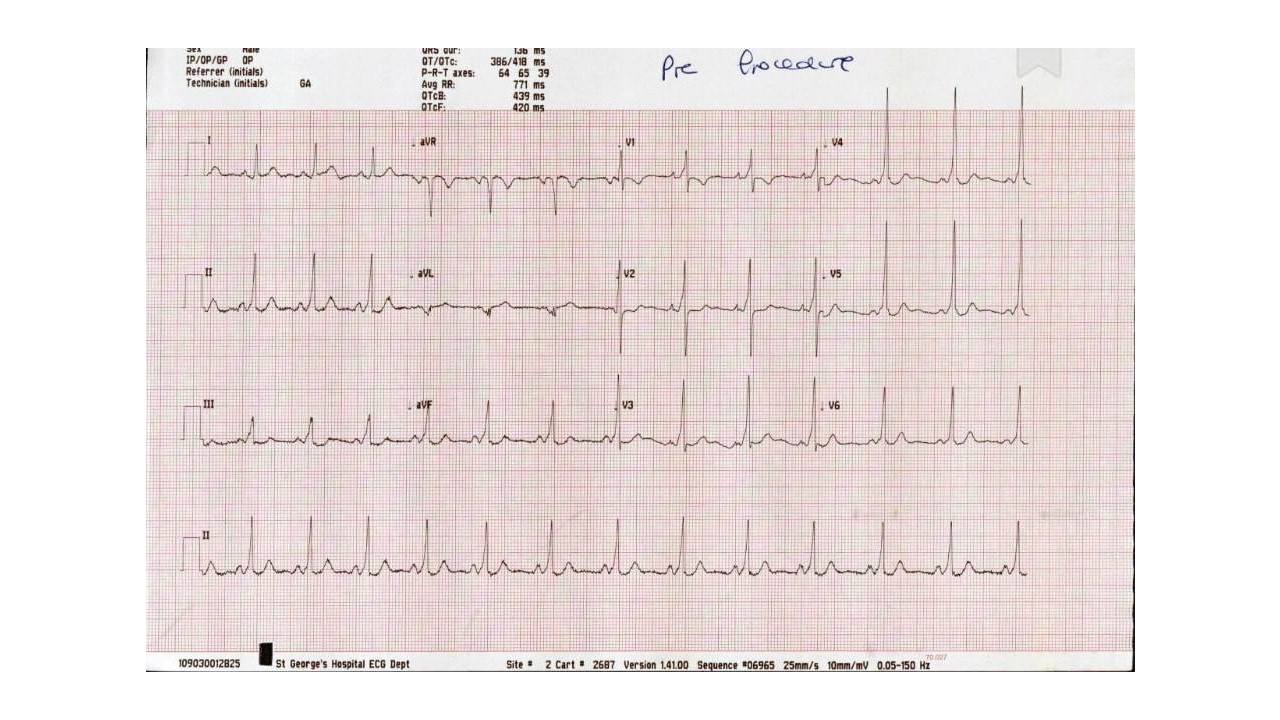

Supplement: Supplementary file 1 — Figure S1 [file CLC-43-897-s001.JPG]

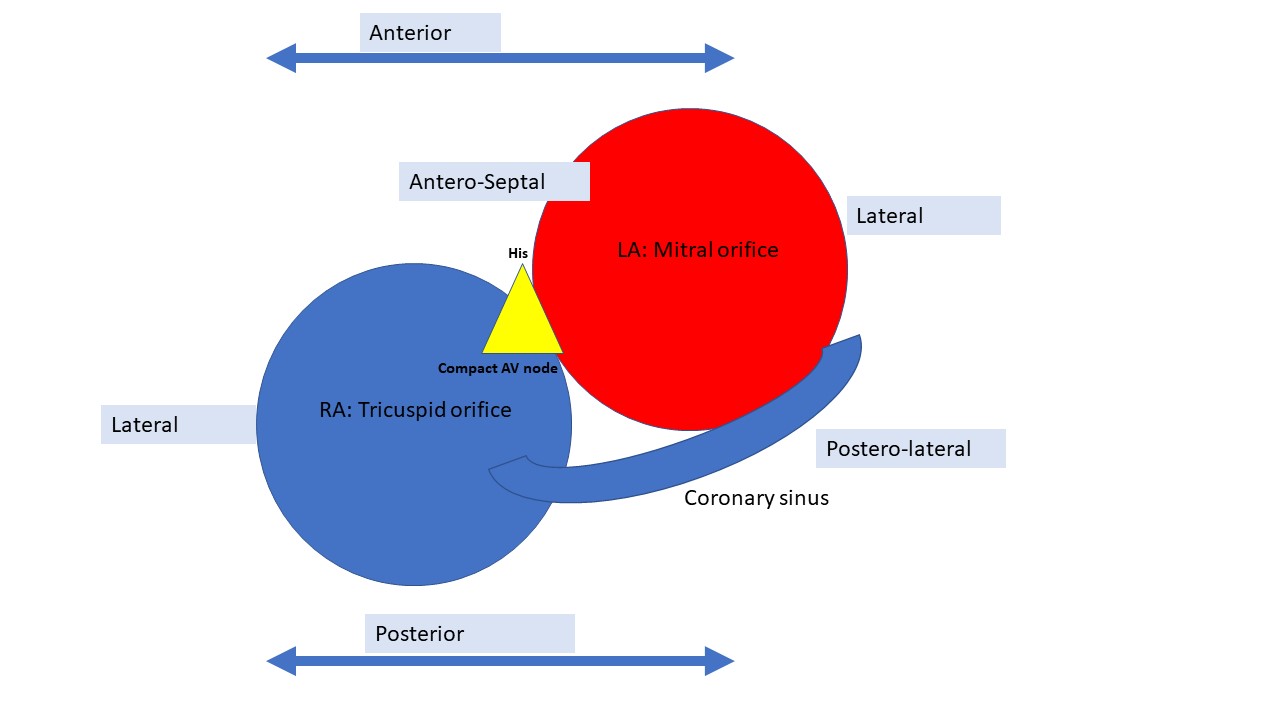

Supplement: Supplementary file 2 — Figure S2 [file CLC-43-897-s002.JPG]
